# Psychological strengths and well-being: Strengths use predicts quality of life, well-being and mental health in autism

**DOI:** 10.1177/13623613221146440

**Published:** 2023-01-13

**Authors:** Emily C Taylor, Lucy A Livingston, Rachel A Clutterbuck, Mitchell J Callan, Punit Shah

**Affiliations:** 1 University of Bath, UK; 2 Cardiff University, UK

**Keywords:** autism, quality of life, strengths, strengths use, well-being

## Abstract

Strengths-based approaches to autism are increasing in research and clinical practice. Such approaches suggest facilitating autistic people to increase the use of their strengths leads to positive outcomes (e.g. improved well-being). However, despite proliferation of strengths-based clinical and educational interventions, these approaches are grounded on several assumptions that remain uninvestigated. Little is known about the specific strengths of autistic people, nor their current knowledge and use of their strengths. Critically, no research has directly tested if autistic people’s strengths knowledge and use is in fact associated with positive outcomes. Conducting an exploratory study, including the first well-powered comparisons of the self-reported strengths, strengths knowledge, and strengths use of matched autistic and non-autistic samples (*N* = 276), we found that autistic and non-autistic participants reported similar strengths. While autistic people reported lower strengths knowledge and use, strengths use in autism strongly predicted better quality of life, subjective well-being, and lower levels of anxiety, depression, and stress. Thus, strength-based approaches and clinical interventions designed to increase strengths use may pose a valuable method for boosting well-being in autism. However, we suggest such approaches should focus on individuals’ strengths more generally, rather than perceived autism-specific abilities.

**Lay abstract:**

It is often suggested that supporting autistic people to identify and use their strengths will lead to positive outcomes. However, little research has explored if this is true. To date, no research has explored whether autistic people already have knowledge of and use their strengths, nor whether increased strengths knowledge and use is linked to good outcomes, such as a better quality of life, well-being and improved mental health. Comparing large samples of autistic and non-autistic people, this study tested these unanswered questions. We found that autistic and non-autistic people reported similar strengths, but autistic people reported less knowledge and use of their strengths compared to non-autistic people. Importantly however, autistic people who reported using their strengths often had better quality of life, well-being and mental health than autistic people who reported using their strengths less frequently. We, therefore, propose that supporting autistic people to use their strengths more often may be a valuable way to boost well-being in this population.

Positive associations between using one’s strengths and well-being is well established (Douglass & Duffy, 2015; Proctor et al., 2011; Wood et al., 2011), with psychological interventions facilitating identification and use of one’s strengths found to promote well-being and reduce depressive symptoms (Ghielen et al., 2018; Schutte & Malouff, 2019). Following their success in the general population, strengths-based interventions have been adopted to promote well-being in clinical populations, including those with depression (e.g. Celano et al., 2017) and anxiety (e.g. Rice et al., 2021).

Interest is growing in strengths-based approaches to support those with lifelong neurodevelopmental conditions, including autism spectrum disorder (ASD, henceforth autism). Historically, autism has been understood in terms of perceived impairments in accordance with medical models and the deficit-based diagnostic criteria (Kapp, 2019; Pellicano & den Houting, 2022). However, autistic people, clinicians and researchers are advocating for a greater appreciation of autistic people’s psychological strengths. For instance, the United Kingdom’s major autism research charity, *Autistica*, published an Action Briefing calling for the adoption of strengths-based approaches to autism in research and clinical practice (Huntley et al., 2019). Strengths-based interventions promoting strengths use in autism are argued to be advantageous for addressing the low quality of life (van Heijst & Geurts, 2015), high rates of co-occurring psychiatric conditions (Hollocks et al., 2019) and low rate of employment (Office for National Statistics, 2016) and university completion (Cage et al., 2020) associated with autism. However, while research and rhetoric on autistic strengths (Clark & Adams, 2020; Meilleur et al., 2015; Russell et al., 2019; Urbanowicz et al., 2019) and strengths-based interventions (M. L. Diener et al., 2016; Lee et al., 2020; Mottron, 2017) is proliferating, the theoretical approach is ill-defined and founded on several assumptions.

First, the definition of strengths in the context of autism has not been explicitly specified in the literature. Therefore, it remains unclear which particular strengths should be incorporated into autism strengths-based interventions. Strengths-based autism research has generally referred to ‘autistic strengths’ (e.g. attention-to-detail, logical thinking) as areas of ability where autistic people perform better than non-autistic people at the group level (e.g. Huntley et al., 2019; Meilleur et al., 2015; Russell et al., 2019). That is, autistic strengths (like autistic difficulties) are not necessarily strengths unique to autism, but strengths that (1) occur more frequently within the autistic than non-autistic population, and more broadly, (2) are present in a large proportion of the autistic population (e.g. Warren et al., 2020).

However, evidence for autistic strengths is largely drawn from qualitative research where strengths were noted by strikingly small samples of autistic people (Carter et al., 2015; Russell et al., 2019), their families (Hastie & Stephens, 2019; Sabapathy et al., 2017; Warren et al., 2020), employers (Dreaver et al., 2019; Lorenz & Heinitz, 2014) and clinicians (de Schipper et al., 2016). Given the qualitative nature of these studies and lack of non-autistic comparisons groups, whether these strengths are more common in autistic versus non-autistic populations remains unclear. Equally, how generalizable such strengths are to all autistic people is unknown. Quantitative cognitive assessments have revealed mixed evidence of autistic strengths, that is, autistic people outperforming non-autistic people (Paola et al., 2021; Robertson & Baron-Cohen, 2017; Van der Hallen et al., 2015). Studies finding evidence of such strengths often fail to replicate, potentially due to limitations common in clinical psychological research, including small underpowered studies, limited case-control group matching (e.g. in cognitive ability), and reliance on observations of clinical traits in the general population. For instance, a recent replication effort has challenged previous research suggesting autistic strengths in logical thinking. Taylor et al. (2022) found no differences between autistic and non-autistic people when conducting well-powered comparisons of large (*N* = 200 ASD) and appropriately matched groups (on age, sex, and general cognitive ability). Much of the variance in logical thinking performance was in fact attributable to general cognitive ability that had not previously been well measured and accounted for.

Without further research quantifying autistic strengths and comparing large autistic and non-autistic samples on these strengths, it remains unclear if they should be specifically incorporated into autism strengths-based interventions. Alternatively, perhaps, a more general focus on strengths (i.e. those common to both autistic and non-autistic populations) would be more beneficial. Indeed, while there is frequently an appreciation that not all autistic people demonstrate specific autistic strengths, and that an individualized approach may be necessary (e.g. Lee et al., 2020; Urbanowicz et al., 2019), proponents of strengths-based approaches nonetheless often discuss these approaches in the context of the broad incorporation of strengths commonly viewed as ‘autistic’ (e.g. attention to detail) into clinical, educational and workplace interventions to improve their efficacy and suitability for autistic people (Huntley et al., 2019). For instance, several strengths-based educational interventions putatively designed to build employment skills have focused on incorporating technology due to ‘the wide recognition of alignment between ICT tasks and the strengths of individuals with ASD’ (Jones et al., 2022, p. 2). Such interventions (see Jones et al., 2022 for review) have largely focused on teaching participants computing skills (3D modelling, coding, robotics) within a set framework designed to leverage autistic people’s skills (attention to detail, technical ability, creativity; see Jones et al. (2021, 2022) for discussion) while also enabling some level of individualization (e.g. tailoring projects to an individual person’s interests).

However, if autism-specific strengths are not evident, continued promotion of ‘autistic strengths’ and interventions incorporating them may do more harm than good, failing to enhance use of individuals’ actual strengths while perpetuating stereotypes to which many autistic people may not conform, creating unrealistic expectations of autistic people; from themselves, employers, and society (see Dawson & Fletcher-Watson, 2021, for discussion of pervasive failures to consider harms in autism interventions). This may also have broader implications for clinical practice, where clinicians are increasingly integrating notions of ‘autistic strengths’ into diagnostic assessments and clinical formulations (Braun et al., 2017; Brown et al., 2021), as well as economic consequences, following global corporations’ engagement in recruitment strategies grounded on ‘autistic strengths’ (e.g. Austin & Pisano, 2017; Cosslett, 2016).

A second issue with strengths-based approaches in autism is that they are based on assumptions that interventions will increase autistic people’s knowledge and use of their strengths (Huntley et al., 2019; Mottron, 2017; Urbanowicz et al., 2019). However, the extent to which autistic people already identify and use their strengths is unclear, with no quantitative research in this area. Given the disproportionate societal emphasis on autism-related ‘deficits’, and lower self-esteem in autism (e.g. Cooper et al., 2017), autistic people are expected to have reduced knowledge of their strengths. Equally, societal constraints may limit strengths use. For example, there may be fewer opportunities for autistic people to use their strengths (e.g. in employment), particularly if their strengths are discouraged due to non-conformity (e.g. intense focus on details). Accordingly, parents report several environmental, developmental, and interpersonal factors present barriers to their autistic child’s strengths use (Clark & Adams, 2020). However, it is possible that autistic people already optimize their strengths use, for example, to compensate for autism-related difficulties (Livingston et al., 2020; Livingston & Happé, 2017). If this is the case, interventions further promoting strengths use may be futile and perhaps detrimental to well-being; strength overuse can result in strengths becoming disadvantageous (e.g. overuse of teamwork strengths leads to dependency), subsequently negatively affecting well-being (see Niemiec, 2019). Given the dearth of empirical research, understanding autistic people’s current levels of strengths knowledge and use is essential to determine whether further enhancement is appropriate.

Finally and most critically, strengths-based approaches suggest increasing strengths use in autistic populations will promote well-being, mental health, quality of life, and employment (Courchesne et al., 2020; Dykshoorn & Cormier, 2019; Huntley et al., 2019). However, this assumes that associations between strengths use and positive outcomes observed in non-autistic populations (e.g. Proctor et al., 2011; Wood et al., 2011) are the same in autistic populations. Autism-related difficulties, combined with potential societal constraints, discrimination, and stigmatism (Han et al., 2022), may limit the extent to which strengths use promotes positive outcomes in autism. Equally, if autistic people possess unique strengths, they may have, or be perceived to have, a different utility and therefore, may not promote positive outcomes in the same way as in non-autistic populations. For instance, in present society, autistic people’s strengths in recognizing patterns may not confer the same promotion of well-being as a non-autistic person’s strengths in social skills. Emerging strength-based interventions implemented in autistic populations have yielded positive outcomes, including in well-being, self-esteem, confidence, and social engagement (e.g. Courchesne et al., 2020; Jones et al., 2022; Lee et al., 2020). However, as an important starting point, these studies have focused on lived experience data in autistic people, therefore necessarily lacking appropriate control comparison groups/interventions, and therefore also limiting outcome attribution to strengths use. Therefore, robust empirical investigation of associations between strengths knowledge and use and positive outcomes in autistic populations is required to determine if programmes designed to harness autistic people’s strengths are an appropriate use of resources.

This research directly tested the aforementioned assumptions underpinning strengths-based approaches to autism. Specifically, we conducted a well-powered comparison of the self-reported strengths of large, well-matched autistic and non-autistic samples. Further, we quantified, for the first time, autistic people’s current strengths knowledge and use, compared to non-autistic people. Finally, we tested whether strengths knowledge and use by autistic people is associated with positive outcomes, including quality of life, subjective well-being and mental health.

## Methods

### Participants

A sample of 276 adults (138 autistic, 138 non-autistic) were recruited via *Prolific.co.* All participants were UK residents and had undergone multiple participant verification processes (see Prolific, 2019). Autistic participants (69 female, 68 male, 1 other), aged 18–63, had clinical diagnoses of an ASD from independent UK or US-based healthcare professionals according to DSM or ICD criteria (American Psychiatric Association, 2013; World Health Organization [WHO], 2019). Participants provided detailed information about their diagnosis (e.g. ASD), diagnosing clinician(s) (e.g. Psychiatrist), and diagnosis location, consistent with previous research recruiting large autistic samples online (e.g. Farmer et al., 2017; Taylor et al., 2022). Diagnoses were confirmed multiple times during a screening process and within the study. All participants had previously participated in autism research (Clutterbuck et al., 2021; Livingston, Shah, & Happé, 2019; Taylor et al., 2022). Non-autistic participants (70 female, 68 male), aged 18–60, did not have autism, nor suspected they were autistic. The autistic and non-autistic groups were age-, sex- and general cognitive ability-matched and there was a large group difference in autistic traits (Table 1). There was a broad range of education and income levels in the sample (Table 1). The groups were comparable in education level: approximately 45% of each group reported completing a level 2 or 3 qualification (e.g. General Certificate of Secondary Education (GCSE) or A-level) and 55% reported completing further education (e.g. bachelor’s degree). The autistic group, however, reported a lower level of income. Specific data on race/ethnicity were not recorded.

**Table 1.** Matching autistic and non-autistic groups.

Measure	Autistic	Non-autistic	Group differences			
			*t*	*p*	*d* [95% CI]	*BF*_10_
Sex (*n* female, male, other)	69, 68, 1	70, 68, 0	–	0.95	–	0.01
Age	29.62 (9.87)	29.47 (9.66)	0.13	0.90	0.02 [−0.22, 0.25]	0.13
General cognitive ability	8.57 (3.59)	8.59 (3.47)	−0.05	0.96	−0.01 [−0.24, 0.23]	0.13
Autistic traits	34.95 (8.51)	19.45 (6.81)	16.71	<0.001	2.01 [1.72, 2.30]	1.39 × 10^40^
Educational attainment	3.66 (1.88)	3.47 (1.85)	0.84	0.40	0.10 [−0.14, 0.34]	0.19
Income	£15.0k (15.6k)	£21.3k (£19.7k)	−2.97	0.003	−0.36 [−0.60, −0.12]	8.33

Values represent means and standard deviations are in parentheses. Independent samples *t* tests are reported, with effect sizes reported as Cohen’s d. General cognitive ability and autistic traits were measured using the International Cognitive Ability Resource (Condon & Revelle, 2014) and Autism-Spectrum Quotient (Baron-Cohen et al., 2001), respectively. Educational attainment was assessed using the 8-point scale of the International Standard Classification of Education (UNESCO Institute for Statistics, 2012), where scores range from 0 (no qualifications) to 7 (Doctorate). CI: confidence interval.

The final sample size gave 80% power to detect at least ‘small-to-medium’ sized effects (α = 0.05, two-tailed) in our group comparisons (*d* = 0.33) and regression analyses (*f*^2^ = 0.040).

### Measures

#### Autism-related psychological strengths

A research-derived list of potential autistic strengths was generated; the 25 most commonly reported psychological strengths from qualitative research investigating autism-related strengths (Clark & Adams, 2020; Colavita, 2014; de Schipper et al., 2016; Dreaver et al., 2019; Russell et al., 2019; Sabapathy et al., 2017; Warren et al., 2020) were identified (see Table 2). Participants were asked ‘To what extent do you agree that the following are *personal strengths of yours*? That is, something that you do well or best’ and responded on a 7-point scale (*Strongly disagree* to *Strongly agree*) for each strength. Scores for each strength range from 1 to 7, with scores 5 or above indicating an endorsement of the trait as a strength.

**Table 2.** Group means and mean differences in autism-related psychological strengths.

Strength	Autistic	Non-autistic	Group differences			
			*t*	*p*	*d* [95% CI]	*BF*_10_
Recognizing patterns	5.62 (1.19)	5.26 (1.23)	2.43	0.016	0.29 [0.06, 0.53]	2.16
Using technology	5.57 (1.44)	5.66 (1.19)	−0.59	0.55	−0.07 [−0.31, 0.17]	0.16
Logical thinking	5.49 (1.52)	5.57 (1.28)	−0.47	0.64	−0.06 [−0.29, 0.18]	0.15
Intelligence	5.47 (1.27)	5.41 (1.01)	0.47	0.64	0.06 [−0.18, 0.29]	0.15
Attention to detail	5.46 (1.51)	5.59 (1.25)	−0.74	0.46	−0.09 [−0.32, 0.15]	0.17
Academic ability	5.36 (1.54)	5.29 (1.30)	0.42	0.67	0.05 [−0.19, 0.29]	0.14
Problem-solving	5.33 (1.47)	5.58 (1.14)	−1.56	0.12	−0.19 [−0.42, 0.05]	0.42
Adherence to routines	5.30 (1.55)	5.01 (1.55)	1.52	0.13	0.18 [−0.05, 0.42]	0.39
Understanding systems	5.17 (1.35)	5.22 (1.23)	−0.33	0.75	−0.04 [−0.28, 0.20]	0.14
Learning new things	5.16 (1.31)	5.58 (1.20)	−2.77	0.006	−0.33 [−0.57, −0.10]	4.96
Repetitive work	5.13 (1.52)	4.92 (1.52)	1.15	0.25	0.14 [−0.10, 0.38]	0.25
Sensory awareness	5.06 (1.53)	5.12 (1.24)	−0.35	0.73	−0.04 [−0.28, 0.19]	0.14
Empathy	4.91 (1.66)	5.66 (1.41)	−4.03	<0.001	−0.49 [−0.72, −0.25]	250.35
Generating ideas	4.77 (1.64)	5.12 (1.35)	−1.93	0.055	−0.23 [−0.47, 0.01]	0.77
Organization	4.71 (1.78)	5.29 (1.55)	−2.89	0.004	−0.35 [−0.59, −0.11]	6.68
Creativity	4.54 (1.84)	4.83 (1.54)	−1.45	0.15	−0.18 [−0.41, 0.06]	0.36
Memory	4.49 (1.71)	4.79 (1.61)	−1.53	0.13	−0.18 [−0.42, 0.05]	0.40
Maths	4.35 (2.02)	4.54 (1.72)	−0.83	0.41	−0.10 [−0.34, 0.14]	0.18
Focus and concentration	4.16 (1.70)	4.78 (1.51)	−3.18	0.002	−0.38 [−0.62, −0.14]	15.15
Communication	4.12 (1.91)	5.24 (1.40)	−5.57	<0.001	−0.67 [−0.91, −0.43]	1.83×10^5^
Artistic ability	3.90 (1.96)	3.80 (1.94)	0.40	0.69	0.05 [−0.19, 0.28]	0.14
Motivation	3.67 (1.79)	4.55 (1.58)	−4.32	<0.001	−0.52 [−0.76, −0.28]	749.69
Physical activity	3.51 (1.95)	4.38 (1.71)	−3.95	<0.001	−0.48 [−0.71, −0.24]	189.03
Musical ability	3.46 (2.02)	3.31 (1.91)	0.61	0.54	0.07 [−0.16, 0.31]	0.16
Social skills	3.30 (1.83)	4.75 (1.58)	−7.09	<0.001	−0.85 [−1.10, −0.61]	6.34 × 10^8^
Number of strengths Endorsed	15.93 (4.91)	17.75 (4.87)	−3.09	0.002	−0.37 [−0.61, −0.13]	11.74

Values represent means and standard deviations are in parentheses. Independent samples *t* tests are reported, with effect sizes reported as Cohen’s *d*. CI: confidence interval.

#### Strengths knowledge

The 8-item Strengths Knowledge Scale (Govindji & Linley, 2007) assessed individuals’ awareness of their strengths, defined as ‘the things that you are able to do well or do best’. Participants responded to items (e.g. ‘I know what I do best’) on a 7-point scale (*Strongly disagree* to *Strongly agree*). Scores range from 8 to 56, with higher scores indicating greater strengths knowledge.

#### Strengths use

The 14-item Strengths Use Scale (Govindji & Linley, 2007) measured self-reported strengths use across a range of settings. Participants responded to items (e.g. ‘I use my strengths everyday’) on a 7-point scale (*Strongly disagree* to *Strongly agree*). Scores range from 14 to 98, with higher scores indicating greater strengths use.

#### Autistic traits

The 50-item Autism-Spectrum Quotient (Baron-Cohen et al., 2001) measured self-reported autistic traits. Participants responded to items (e.g. ‘I find social situations easy’), on a 4-point scale (*Definitely agree* to *Definitely disagree*). Scores range from 0 to 50, with higher scores indicating more autistic traits.

#### General cognitive ability

The 16-item version of the International Cognitive Ability Resource (ICAR; Condon & Revelle, 2014) assessed general cognitive ability. This well-validated measure was purposefully designed for online use, strongly correlates with in-person intelligence tests (e.g. Wechsler Adult Intelligence Scale; Condon & Revelle, 2014; Dworak et al., 2020; Young & Keith, 2020), and has been used previously in autism research (e.g. Clutterbuck et al., 2021; Farmer et al., 2017; Taylor et al., 2022). Scores range from 0 to 16, with higher scores indicating higher cognitive ability.

#### Quality of life

The 26-item WHO Quality of Life Instrument, Abbreviated Version (WHOQOL-BREF; WHOQOL Group, 1998) assessed self-reported quality of life in four separate domains: physical health, psychological health, social relationships, and environment. Domain scores range from 4 to 20, with higher scores indicating better quality of life. Autistic participants also completed the WHOQOL-Disabilities Module (Power & Green, 2010) and Autism-Specific QoL (ASQoL; McConachie et al., 2018), which supplement the WHOQOL-BREF for a comprehensive understanding of quality of life in autistic populations. Thus, for the autistic group, an additional composite autism quality of life score was calculated, summing standardized WHO-QOL-Disabilities module and ASQoL scores.

#### Subjective well-being

Following previous research, subjective well-being was measured as a composite of life satisfaction, positive affect, and negative affect (E. Diener & Lucas, 1999; Govindji & Linley, 2007; Proctor et al., 2011). The 5-item Satisfaction with Life Scale (E. Diener et al., 1985) measured self-reported global life satisfaction. Participants responded to items (e.g. ‘I am satisfied with my life’) using a 7-point scale (*Strongly disagree* to *Strongly agree*). Scores range from 5 to 35. The Positive and Negative Affect Schedule (Watson et al., 1988) measured self-reported positive and negative affect on two 10-item subscales. Participants indicated to what extent they felt each affect (e.g. excited, distressed) in the past week on a 5-point scale (*Very slightly or Not at all* to *Extremely*). Subscale scores range from 10 to 50. To calculate subjective well-being scores (as in e.g. Govindji & Linley, 2007), standardized negative affect scores were subtracted from the sum of standardized life-satisfaction and positive affect scores. Higher scores indicate greater subjective well-being.

#### Mental health

The 21-item Depression Anxiety and Stress Scale (DASS-21; Lovibond & Lovibond, 1995) assessed self-reported mental health problems, quantifying depression, anxiety and stress symptoms in three subscales. Participants reported the frequency of experiencing symptoms in the last week on a 4-point scale (*Not at all* to *Most of the time*). This measure has previously been validated and used in autism research (e.g. Park et al., 2020; Taylor et al., 2021). Subscale scores range from 0 to 42, with higher scores indicating more mental health symptoms.

### Procedure

Clearance was received from the local ethics committee, and participants gave informed consent when starting the study. Measures were presented in a randomized order. To ensure the research-derived list of strengths did not prime participant’s perceptions of their strengths knowledge and use, the Autism-Related Psychological Strengths measure was presented after the Strengths Knowledge and Strengths Use measures.

### Community involvement

Following participatory autism research guidelines (Fletcher-Watson et al., 2019), the study was co-developed with autistic adults of different backgrounds, ages, and genders. This ensured our aims were relevant to the autism community and that the study procedure was appropriate (e.g. used suitable language). Specifically, autistic people were involved in the development of the research question, study design, and interpretation of the findings.

## Results

All measures showed acceptable-to-excellent internal consistency, with comparable internal consistency within the autistic and non-autistic groups (Table S1). Most notably, the Strengths Knowledge and Strengths Use scale, which had not previously been used in autistic samples, showed excellent internal consistency, with α > 0.9 in both the autistic and non-autistic groups.

### Differences in self-reported strengths

The autistic group endorsed (scored 5 or above) fewer autism-related psychological strengths than the non-autistic group (Table 2). On average, the autistic group endorsed 15.93 strengths (*SD* = 4.91), whereas the non-autistic group endorsed 17.75 (*SD* = 4.87). The autistic group endorsed recognizing patterns more strongly than the non-autistic group. Contrastingly, the non-autistic group more strongly endorsed learning new things, empathy, organization, focus, communication, motivation, physical activity and social skills. There were no group differences on the other strengths, with Bayes Factors supporting the null result (see Supplementary Materials for details on Bayesian analyses).

### Differences in strengths knowledge and use

Strengths knowledge was significantly lower in the autistic (*M* = 37.22, *SD* = 8.98) than non-autistic group (*M* = 40.70, *SD* = 7.74), *t*(274) = −3.45, *p* < 0.001, *d* = 0.42, *BF*_10_ = 34.65. Strengths use was also significantly lower in the autistic (*M* = 62.73, *SD* = 15.84) than non-autistic group (*M* = 69.91, *SD* = 12.45), *t*(274) = −4.18, *p* < 0.001, *d* = 0.50, *BF*_10_ = 452.53. Group differences in strengths use remained after accounting for strengths knowledge, *F* (1,273) = 5.49, *p* = 0.020, *η*_p_^2^ = 0.020, *BF*_incl_ = 1.87.

### Associations between strengths knowledge, strengths use, and positive outcomes

**Figure 1.**
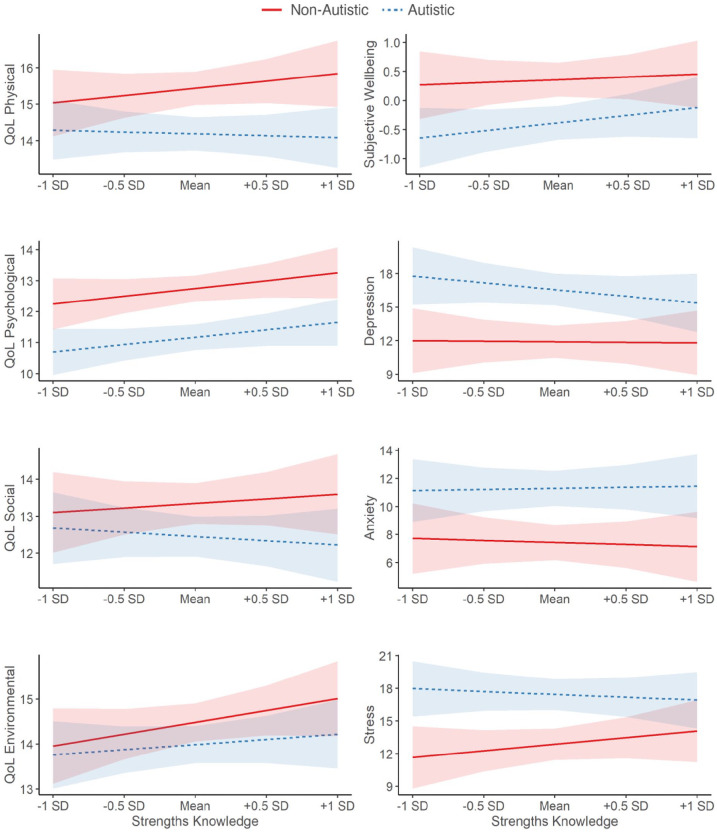
Relationships between strengths knowledge and quality of life (QoL), subjective well-being, and mental health symptoms, as a function of autism. Modelled relationships are after accounting for strengths use, age, sex, and general cognitive ability, as well as their interactions with autism. All predictors were mean-centred. 95% confidence intervals are depicted. Results of the full moderation analyses are reported in Tables S3 and S4.

**Figure 2.**
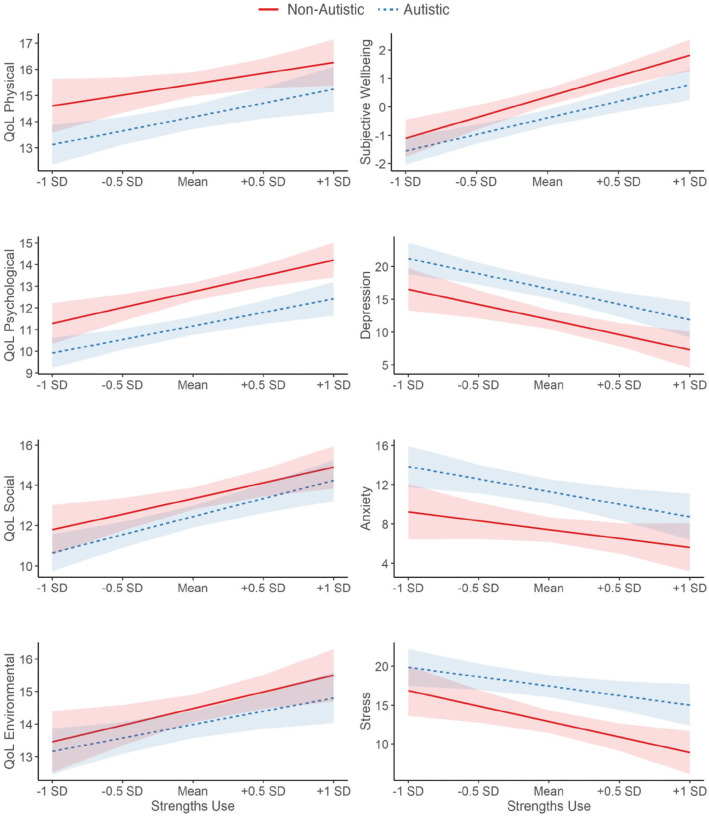
Relationships between strengths use and quality of life (QoL), subjective well-being and mental health symptoms, as a function of autism. Modelled relationships are after accounting for strengths knowledge, age, sex, and general cognitive ability, as well as their interactions with autism. All predictors were mean-centred. 95% confidence intervals are depicted. Results of the full moderation analyses are reported in Tables S3 and S4.

The autistic group reported lower quality of life and subjective well-being, and more mental health symptoms than the non-autistic group (Table S2). Regression analyses examined the contributions of strengths knowledge, strengths use, and autism to these outcomes, while accounting for age, sex and general cognitive ability (Tables S3 and S4). Interactions between autism and each of the predictors were modelled. Strengths knowledge was only a predictor of better quality of life in the psychological domain (Figure 1). Strengths use was a large predictor of better quality of life across all four domains, higher subjective well-being, and fewer mental health symptoms (Figure 2). Autism had a smaller, opposite effect, predicting lower quality of life (except in the environmental domain), lower subjective well-being, and more mental health symptoms. Critically, across the analyses, interactions between autism and strengths knowledge or strengths use were not significant, and inclusion Bayes Factors suggested more evidence for the null hypothesis (i.e. interaction terms should not be included in the final model; Tables S3 and S4). Thus, the identified (null) relationships between strengths knowledge, strengths use, and the outcomes did not differ between the autistic and non-autistic groups (Figures 1 and 2).

#### Quality of life within the autistic group

An additional regression analysis, conducted within the autistic group, showed – after accounting for age, sex and general cognitive ability – strengths use, but not strengths knowledge, was a significant positive predictor of the autism-specific quality of life measure (Table S5). Repeating the analysis with autistic trait scores included as a proxy for autism severity, revealed the same pattern (Table S6). Autistic traits were a negative predictor of quality of life, but interactions between autistic traits and strengths knowledge and use were not significant. Thus, autism severity did not influence the positive association between strengths use and quality of life in autism.

## Discussion

Although strengths-based approaches to autism have garnered significant attention from the autism community, researchers, and clinicians, they are grounded in several assumptions that remain to be tested. In the largest empirical examination of strengths in autism, we quantified, for the first time, the self-reported strengths of a diverse autistic sample. Well-powered comparisons of matched autistic and non-autistic groups showed little evidence for autism-specific strengths. However, autistic people reported less knowledge and use of their strengths. Critically, similarly to non-autistic people, strengths use by autistic people was strongly associated with positive outcomes, including better quality of life, subjective well-being, and mental health. Together, our results suggest that strength-based approaches promoting strengths use may be an advantageous, under-researched method for enhancing well-being in autistic populations. However, clinical and educational interventions should focus on promoting strengths use more generally, rather than narrowly concentrating on ‘autistic strengths’.

We found few differences between autistic and non-autistic people’s endorsement of ‘autistic strengths’ previously identified in autism research. Only one strength (pattern recognition) was endorsed more by autistic participants, challenging the idea that autistic people, at the group level, possess autism-specific strengths. In fact, 8 of the 25 characteristics identified as autism-related strengths in previous research, were endorsed more by the non-autistic than autistic group, suggesting an incorrect characterization of these traits. This clarifies previous qualitative literature where the idea of ‘autistic strengths’ has proliferated without direct comparisons between autistic and non-autistic people (e.g. de Schipper et al., 2016; Russell et al., 2019; Sabapathy et al., 2017). Given our results, we recommend moving away from the term ‘autistic strengths’ and their specific incorporation into interventions, towards acknowledging the many diverse strengths autistic people have but share with their non-autistic peers. Following cognitive heterogeneity in autism (Masi et al., 2017), there is likely large variability in autistic people’s strengths. Thus, generalizations regarding strengths of autistic populations, as a whole, are likely inaccurate and may promote stereotypes of autistic people. Highlighting ‘autistic strengths’, for instance, to promote employment of autistic people (e.g. Austin & Pisano, 2017; Cosslett, 2016), while well intentioned, likely does more harm than good to autistic people who do not demonstrate enhanced performance in these domains. Likewise, clinicians incorporating ‘autistic strengths’ into diagnostic assessments and clinical formulations may inappropriately attribute someone’s abilities to an autism diagnosis. Appreciating similarities between autistic and non-autistic people, and their diverse but not necessarily unique strengths, may be a more beneficial approach. This approach may help to build cohesion between autistic and non-autistic populations, improving attitudes towards those with clinical conditions (Hanel et al., 2019; Taylor et al., 2022), while addressing unhelpful rhetoric that autistic populations must offer unique strengths to make contributions to society (Pellicano & den Houting, 2022).

Our study was the first to quantify autistic people’s knowledge and use of their strengths. The finding of lower strengths knowledge and use in the autistic group aligns with the assumptions of strengths-based approaches, suggesting the potential for enhancement in autistic populations (e.g. Dykshoorn & Cormier, 2019; Huntley et al., 2019). In non-autistic populations, identification and encouragement of strengths by others is key to building strengths knowledge (e.g. Allan et al., 2021). Given the long-standing deficit approach to autism, external signals from society, clinicians, caregivers, and teachers may be orientated towards identifying and supporting autistic people’s difficulties, limiting positive cues that aid strength identification. Equally, non-autistic people may be less able to recognize and support strengths in autistic people, particularly if strengths are masked by difficulties. Together this may contribute to lower self-efficacy, self-esteem, and self-confidence in autism, which could further limit strengths knowledge.

Consistent with previous literature (e.g. Govindji & Linley, 2007), lower strengths use in autism was partly explained by reduced strengths knowledge. However, as lower strengths use was observed after accounting for strengths knowledge, other factors (e.g. few employment opportunities matching autistic individuals’ skillset), contribute to lower strengths use in autism. This aligns with Clark and Adams’ (2020) finding that autistic children experience several barriers to engaging their strengths. Exploring if similar barriers are experienced by autistic adults would be highly valuable. In addition to those faced by autistic children, several unique barriers may emerge in adulthood, including those associated with work and complex social relationships. Understanding whether internal (e.g. ability to identify opportunities to use strengths) or external (e.g. societal attitudes) factors are larger contributors to strengths use by autistic people will be critical to determining how to increase strengths use; interventions building strengths use may have little impact if factors outside the individual’s control limit real-world strengths implementation.

With no previous research in this area, it was unclear if strengths knowledge or use by autistic people is linked to positive outcomes. We found strengths use by autistic people was associated with better quality of life, well-being, and mental health. In fact, strengths use consistently made larger and opposite contributions to these outcomes compared to autism itself; autistic people with high strengths use had better outcomes than non-autistic people with low strengths use. Thus, strengths use could help overcome the lower quality of life and well-being associated with autism and may serve as an important protective factor, preventing promotion of co-occurring psychiatric conditions (see McCrimmon & Montgomery, 2014). Previous research in non-autistic populations suggests strengths use increases well-being through building individuals’ feelings of self-worth, inducing positive affect and self-esteem (Douglass & Duffy, 2015). Given the similarities between autistic and non-autistic people in this research, an equivalent mechanism may underpin the identified association in autism, however, this needs empirical testing. Future research, particularly with longitudinal designs, should explore these mechanisms, establishing the directionality and protective effects of strengths use in autistic populations.

Our findings have important clinical implications, supporting proposals that building strengths knowledge and use in autistic populations is a valuable, presently under-researched, approach to boost quality of life and well-being. Following the similar relationships between strengths use and well-being in autistic and non-autistic people, it should be explored if well-established efficacious interventions for non-autistic populations (e.g. interventions facilitating identification of opportunities for strengths use; see Ghielen et al., 2018; Schutte & Malouff, 2019 for reviews) show similar positive effects in autistic populations. While programmes may require adapting to suit autistic people’s needs, developing existing programmes is an efficient, resource-conscious approach. Arguably, such programmes would have a higher chance of efficacious outcomes than emerging autism-specific strengths-based interventions grounded on ‘autistic strengths’, which require significant commitments from autistic people with unknown outcomes. However, given the limited understanding of the factors contributing to lower strengths use in autism, barriers to engagement may not be presently addressed in either autism-specific or non-autistic interventions. Thus, further research into these barriers is vital to ensure interventions appropriately target the source of difficulties. Without doing so, strengths-based approaches may have limited, or even detrimental, effects for autistic people’s mental health and well-being.

### Strengths and limitations

The present research has numerous strengths, including the comparison of large, well-matched autistic and non-autistic samples. Recruiting both groups online through the same source increased internal validity, reducing group differences resulting from recruiting autistic and non-autistic people through different methods (e.g. databases of autistic participants vs, social media), which commonly occurs in strengths-based autism research (e.g. Brosnan et al., 2017; Remington & Fairnie, 2017). This approach also enabled recruitment of participants less likely to participate in in-person research (e.g. due to resource constraints, anxiety; see Livingston, Carr, & Shah, 2019), resulting in a heterogeneous sample of autistic people with diverse educational and employment backgrounds. Further, using a research-derived list of ‘autistic strengths’ allowed for the quantification and direct comparison of autistic and non-autistic people on the most widely cited autistic strengths. This also facilitated autistic people who may experience difficulties in free recall of their strengths. Finally, by using multiple outcome measures, we have shown that strengths use in autism is associated with positive outcomes across well-validated measures recommended by WHO, sensitive autism-specific measures of quality of life, and mental health and well-being outcomes that of most importance to autistic people (Crane et al., 2019).

There were limitations to be addressed. The online research methods and study design may have precluded the participation of people with reduced access to the Internet, additional support needs, and those with intellectual disability. Conducting research online also limited the use of neurocognitive measures of strengths and thus, the study was largely reliant of self-report tools. Measuring people’s self-reported strengths and strengths use may have been skewed by individuals’ perception or societal norms of whether traits are considered a strength. Resultantly, autistic people may have and use many of the listed strengths but may not perceive or report them as such. For instance, ‘sensory awareness’ may not be perceived as a strength given its associations with several difficulties (e.g. hypersensitivity to lights). Further, autism is associated with metacognitive difficulties (Brosnan et al., 2016; Furlano & Kelley, 2020). Thus, autistic people may experience difficulties accurately reporting their strengths. Indeed, this may partly underpin the lower strengths knowledge in autism. However, our self-report approach was comparable to previous autism strengths-based literature, where autistic adults demonstrated sufficient metacognitive insight to describe their strengths (e.g. Russell et al., 2019). Further, self-report tools, widely used in autism research, have been found to be valid and reliable in autistic populations, correlating well with performance on cognitive measures (e.g. Clutterbuck et al., 2021). Nonetheless, research objectively measuring strengths, their use, and associated outcomes in autism should be conducted. Indeed, exploring how different strengths, or types of strengths, link to strength use and well-being in both autistic and non-autistic populations will be a particularly important avenue for future research. Equally, moving forward, a more nuanced consideration of autism-related strengths within the context of autism-related difficulties would be beneficial. Indeed, strengths and difficulties have largely been considered independently in autism-related research, though they are highly likely to be interdependent – potentially as ‘double-edged swords’ (see also, Russell et al., 2019 for discussion).

We did not characterize or account for other clinical and neurodevelopmental conditions in either the autistic or non-autistic group. ADHD, which frequently co-occurs with autism (Hollocks et al., 2019), is thought to be linked with unique strengths and strength uses (e.g. hyper-focus; Sedgwick et al., 2018). Thus, higher rates of ADHD in our autistic group could contribute to the group differences in strengths knowledge and use and may potentially moderate their associations with positive outcomes. Equally, while we explored whether strengths use predicted mental health symptoms, that is, in accordance with positive psychology models of the relationships between strength use and psychological well-being, a complex, bidirectional relationship is likely, whereby depression and anxiety also contribute to lower strengths knowledge and use. In future, exploring how mental health conditions are linked to strengths use and well-being will be critical to inform the design of strengths-based interventions and their appropriateness for autistic individuals with and without co-occurring conditions. For instance, longitudinal explorations of the directionality between strength use and mental health, or a replication of the present study where autistic and non-autistic groups are matched on levels of anxiety and depressive symptoms, might be useful. Finally, we did not collect information regarding ethnicity and race. While there was a high level of variance in education levels in our samples, probably more so than in classical lab-based studies on autism, all participants were generally well-educated and thus may not be representative of the wider population. Thus, the effects of these demographic variables on our findings require further investigation.

## Conclusion

This research presents quantitative insights into autistic people’s strengths, highlighting many similarities in the strengths reported by autistic and non-autistic people. Critically, we found that strengths use in autism is positively linked to well-being and mental health. We, therefore, suggest clinical and educational interventions designed to increase the lower strengths use observed in autism, may present an advantageous, currently underappreciated tool for promoting well-being and mental health in autistic populations. Moving forward, however, we recommend that strengths-based approaches focus on individuals’ strengths more generally, rather than previously characterized ‘autistic strengths’ that are currently not well evidenced in empirical research. Finally, we highlight that building understanding of the barriers autistic adults experience to using their strengths will be critical to ensure appropriate support for autistic people’s strengths use and well-being.

## Supplemental Material

sj-docx-1-aut-10.1177_13623613221146440 – Supplemental material for Psychological strengths and well-being: Strengths use predicts quality of life, well-being and mental health in autism Click here for additional data file. Supplemental material, sj-docx-1-aut-10.1177_13623613221146440 for Psychological strengths and well-being: Strengths use predicts quality of life, well-being and mental health in autism by Emily C Taylor, Lucy A Livingston, Rachel A Clutterbuck, Mitchell J Callan and Punit Shah in Autism
