# Supplementary material for: Psychological strengths and well-being: Strengths use predicts quality of life, well-being and mental health in autism
Source: Autism. 2023 Jan 13;27(6):1826–39. doi: 10.1177/13623613221146440 (PMC10375006; doi:10.1177/13623613221146440)
Supplement: sj-docx-1-aut-10.1177_13623613221146440 – Supplemental material for Psychological strengths and well-being: Strengths use predicts quality of life, well-being and mental health in autism [file sj-docx-1-aut-10.1177_13623613221146440.docx]

**Supplementary Material**

Psychological Strengths and Wellbeing: Strengths Use Predicts Quality of Life, Wellbeing and Mental Health in Autism

**Bayesian Analyses**

Conventional statistical methods (i.e., frequentist analyses) involve null-hypothesis significance testing. However, there is a growing appreciation of the shortcomings of this approach, particularly when interpreting null effects (Wagenmakers, 2007). While it is possible to reject the null hypothesis using these tests, it is difficult to find support for the null hypothesis.

Bayesian inference is an alternative statistical approach that addresses this issue. The Bayes Factor (BF_10_) is a continuous measure of evidence describing the probability of obtaining the data under an explicitly outlined alternative hypothesis (H_1_) relative to the probability under the null hypothesis (H_0_): BF_10_ = Pr (D|H_1_) / Pr (D|H_0_). A BF_10_ of 0.2, for example, indicates that the data observed are approximately 5 times more likely under the null than alternative hypothesis. Following commonly accepted interpretations of BF_10_ (see Wagenmakers et al., 2011), this would indicate ‘substantial’ evidence for the null hypothesis.

For all our analyses, we conducted Bayesian equivalent tests to determine if the data provided more evidence in support of the null vs. alternative hypothesis. When directly comparing the autistic and non-autistic groups, we conducted Bayesian t-tests to quantify the support for the two-tailed alternative hypothesis (*H_1_* = autistic and non-autistic groups have different mean scores) relative to the null hypothesis (*H_0_* = autistic and non-autistic groups do not have different mean scores). For the regression analyses, we quantified the support for the two-tailed alternative hypothesis (*H_1_* = the predictor explains unique variance in the outcome, so should be included in the final model) relative to the null hypothesis (*H_0_* = the predictor does not explain unique variance in the outcome, so should not be included in the final model) for each of the predictors. As interaction terms were included in the model, we report inclusion Bayes Factors across matched models (i.e., comparing models containing the effect to equivalent models stripped of the effect), in accordance to recommendations by Mathôt (2017) and JASP (JASP Team, 2021). This is to avoid conflation of Bayes Factors for main effects and interaction terms.

All analyses were conducted in JASP 0.16 (JASP Team, 2021). Bayesian modelling requires a specified prior probability distribution for the alternative hypothesis model parameters. *JASP* (JASP Team, 2021) provides default, broadly applicable, uninformative priors (see Quintana & Williams, 2018) that can be used when no prior knowledge regarding the hypotheses is available. We opted to use these priors throughout our analyses, given that there is no well-established literature in this domain. Further, for multivariate analyses, we specified that the prior distribution of the models was uniform (i.e., each of the models were equally likely). This approach to Bayesian analysis is recommended by Rouder et al. (2012) (see also, Wagenmakers et al. (2018) and Quintana and Williams (2018) for recent information regarding Bayesian inference).

**Supplementary Tables**

Table S1

*Measures and their Internal Consistency within the Autistic and Non-Autistic Groups.*

| Measure | Autistic | | Non-Autistic | |
| --- | --- | --- | --- | --- |
|  | ω | α | ω | α |
| Strengths Knowledge | .92 | .92 | .92 | .92 |
| Strengths Use | .94 | .94 | .93 | .93 |
| Satisfaction with Life | .87 | .87 | .90 | .89 |
| Positive Affect | .91 | .90 | .92 | .92 |
| Negative Affect | .91 | .90 | .90 | .90 |
| WHOQOL-BREF Physical | .86 | .86 | .80 | .79 |
| WHOQOL-BREF Psychological | .85 | .84 | .84 | .83 |
| WHOQOL-BREF Social | .72 | .69 | .71 | .69 |
| WHOQOL-BREF Environmental | .79 | .79 | .83 | .83 |
| DASS-21 Depression | .92 | .91 | .90 | .89 |
| DASS-21 Anxiety | .86 | .86 | .82 | .81 |
| DASS-21 Stress | .86 | .86 | .87 | .87 |
| WHOQOL Disabilities Module | .83 | .80 |  |  |
| Autism-Specific QoL | .69 | .70 |  |  |

Table S2

*Autistic and Non-Autistic Group Means and Mean Differences*

| Measure | Autistic | Non-Autistic | Group Differences | | | |
| --- | --- | --- | --- | --- | --- | --- |
|  |  |  | *t* | *p* | *d* [95% CI] | *BF_10_* |
| Strengths Knowledge | 37.22 (8.98) | 40.70 (7.74) | -3.45 | <.001 | -0.42 [-0.65, -0.18] | 34.65 |
| Strengths Use | 62.73 (15.84) | 69.91 (12.45) | -4.18 | <.001 | -0.50 [-0.74, -0.26] | 452.53 |
| Subjective Wellbeing | -0.73 (2.37) | 0.73 (2.09) | -5.43 | <.001 | -0.65 [-0.90, -0.41] | 9.51×10^4^ |
| QoL Physical | 13.90 (3.33) | 15.72 (2.54) | -5.09 | <.001 | -0.61 [-0.85, -0.37] | 1.98×10^4^ |
| QoL Psychological | 10.78 (3.12) | 13.21 (2.86) | -6.75 | <.001 | -0.81 [-1.06, -0.57] | 8.95×10^7^ |
| QoL Social | 12.08 (3.68) | 13.78 (3.32) | -4.03 | <.001 | -0.49 [-0.72, -0.25] | 251.83 |
| QoL Environmental | 13.70 (2.75) | 14.84 (2.64) | -3.52 | <.001 | -0.42 [-0.66, -0.19] | 43.63 |
| Depression | 17.94 (10.99) | 10.78 (8.41) | 6.08 | <.001 | 0.73 [0.49, 0.98] | 2.35×10^6^ |
| Anxiety | 11.94 (8.89) | 6.94 (6.40) | 5.36 | <.001 | 0.65 [0.40, 0.89] | 6.85×10^4^ |
| Stress | 18.20 (9.27) | 12.16 (8.24) | 5.72 | <.001 | 0.69 [0.45, 0.93] | 3.93×10^5^ |

*Note.* Standard deviations are in parentheses. Independent samples t-tests are reported, with effect sizes reported as Cohen’s *d*. Robust t-tests and Mann-Whitney U tests produced the same pattern of results. The same pattern was also observed with ANCOVA comparing autistic and non-autistic groups while also accounting for age, sex and general cognitive ability.

Table S3

*Regression Analyses Predicting WHOQOLBREF Quality of Life Domains across Both the Autistic and Non-Autistic Groups*

|  | Physical | | | |  | Psychological | | | |  | Social | | | |  | Environmental | | | |
| --- | --- | --- | --- | --- | --- | --- | --- | --- | --- | --- | --- | --- | --- | --- | --- | --- | --- | --- | --- |
| Predictors | *B* | *SE_B_* | *β* | *BF_incl_* |  | *B* | *SE_B_* | *β* | *BF_incl_* |  | *B* | *SE_B_* | *β* | *BF_incl_* |  | *B* | *SE_B_* | *β* | *BF_incl_* |
| Autism | -1.26 | 0.33 | -0.21** | 115.33 |  | -1.58 | 0.30 | -0.25** | 3.94×10^4^ |  | -0.90 | 0.39 | -0.13* | 1.91 |  | -0.50 | 0.30 | -0.09 | 0.61 |
| Strengths Knowledge | 0.02 | 0.03 | 0.05 | 0.21 |  | 0.06 | 0.03 | 0.15* | 0.61 |  | 0.00 | 0.04 | 0.00 | 0.13 |  | 0.04 | 0.03 | 0.14 | 0.36 |
| Strengths Use | 0.06 | 0.02 | 0.31** | 476.30 |  | 0.09 | 0.02 | 0.42** | 1.51×10^6^ |  | 0.11 | 0.02 | 0.47** | 3.64×10^6^ |  | 0.06 | 0.02 | 0.34** | 1110.10 |
| Age (years) | -0.02 | 0.02 | -0.07 | 0.36 |  | 0.03 | 0.02 | 0.10* | 1.46 |  | 0.00 | 0.02 | -0.01 | 0.13 |  | -0.01 | 0.02 | -0.03 | 0.15 |
| Sex | 0.98 | 0.33 | 0.16* | 17.35 |  | 0.52 | 0.30 | 0.08 | 0.69 |  | -0.24 | 0.39 | -0.03 | 0.15 |  | -0.02 | 0.30 | 0.00 | 0.13 |
| General Cognitive Ability | -0.10 | 0.05 | -0.11* | 1.74 |  | -0.08 | 0.04 | -0.08 | 0.67 |  | -0.03 | 0.06 | -0.03 | 0.15 |  | -0.03 | 0.04 | -0.03 | 0.17 |
| Autism * Strengths Knowledge | -0.06 | 0.06 | -0.08 | 0.33 |  | 0.00 | 0.06 | 0.00 | 0.18 |  | -0.06 | 0.08 | -0.07 | 0.20 |  | -0.04 | 0.06 | -0.05 | 0.38 |
| Autism * Strengths Use | 0.02 | 0.04 | 0.04 | 0.20 |  | -0.02 | 0.03 | -0.03 | 0.19 |  | 0.02 | 0.05 | 0.03 | 0.14 |  | -0.01 | 0.03 | -0.04 | 0.33 |
| Autism * Age (years) | -0.03 | 0.03 | -0.05 | 0.38 |  | 0.01 | 0.03 | 0.02 | 0.14 |  | 0.00 | 0.04 | 0.01 | 0.16 |  | 0.00 | 0.03 | 0.00 | 0.19 |
| Autism * Sex | 1.69 | 0.65 | 0.14* | 4.05 |  | 1.37 | 0.59 | 0.11* | 1.97 |  | 0.28 | 0.78 | 0.02 | 0.20 |  | 0.67 | 0.60 | 0.06 | 0.31 |
| Autism * General Cognitive Ability | -0.08 | 0.09 | -0.05 | 0.34 |  | -0.04 | 0.09 | -0.02 | 0.16 |  | -0.03 | 0.11 | -0.01 | 0.17 |  | -0.10 | 0.09 | -0.07 | 0.40 |
| Model Statistics | *F*(11, 263) = 9.58  *p* <.001, *R^2^* = .29 | | | |  | *F*(11, 263) = 21.28  *p* <.001, *R^2^* = .47 | | | |  | *F*(11, 263) = 8.70  *p* <.001, *R^2^* = .27 | | | |  | *F*(11, 263) = 7.57  *p* <.001, *R^2^* = .24 | | | |

*Note.* Predictors are mean centered, with interaction terms calculated using the centered variables. Males = 1 and Females = 0 when coding participant sex.

* *p* < .05, ** *p* < .001

Table S4

*Regression Analyses Predicting Subjective Wellbeing and Mental Health Symptoms across Both the Autistic and Non-Autistic Groups*

|  | Subjective  Wellbeing | | | |  | Depression | | | |  | Anxiety | | | |  | Stress | | | |
| --- | --- | --- | --- | --- | --- | --- | --- | --- | --- | --- | --- | --- | --- | --- | --- | --- | --- | --- | --- |
| Predictors | *B* | *SE_B_* | *β* | *BF_incl_* |  | *B* | *SE_B_* | *β* | *BF_incl_* |  | *B* | *SE_B_* | *β* | *BF_incl_* |  | *B* | *SE_B_* | *β* | *BF_incl_* |
| Autism | -0.75 | 0.21 | -0.16** | 45.02 |  | 4.68 | 1.04 | 0.23** | 1426.40 |  | 3.88 | 0.91 | 0.24** | 903.72 |  | 4.57 | 1.03 | 0.25** | 2042.89 |
| Strengths Knowledge | 0.02 | 0.02 | 0.08 | 0.21 |  | -0.08 | 0.10 | -0.06 | 0.19 |  | -0.01 | 0.09 | -0.01 | 0.25 |  | 0.04 | 0.10 | 0.04 | 0.18 |
| Strengths Use | 0.09 | 0.01 | 0.56** | 1.60×10^11^ |  | -0.32 | 0.06 | -0.45** | 1.42×10^6^ |  | -0.15 | 0.05 | -0.27* | 84.51 |  | -0.22 | 0.06 | -0.35** | 661.12 |
| Age (years) | 0.03 | 0.01 | 0.11* | 2.23 |  | -0.08 | 0.05 | -0.07 | 0.30 |  | -0.12 | 0.05 | -0.14* | 4.72 |  | -0.03 | 0.05 | -0.03 | 0.18 |
| Sex | 0.40 | 0.21 | 0.08 | 0.81 |  | -1.85 | 1.02 | -0.09 | 0.72 |  | -2.73 | 0.89 | -0.17* | 18.00 |  | -3.36 | 1.02 | -0.18* | 18.37 |
| General Cognitive Ability | -0.03 | 0.03 | -0.05 | 0.26 |  | -0.14 | 0.15 | -0.05 | 0.17 |  | -0.24 | 0.13 | -0.10 | 1.08 |  | -0.12 | 0.15 | -0.05 | 0.20 |
| Autism * Strengths Knowledge | 0.02 | 0.04 | 0.04 | 0.14 |  | -0.13 | 0.20 | -0.05 | 0.21 |  | 0.05 | 0.17 | 0.03 | 0.23 |  | -0.20 | 0.20 | -0.09 | 0.25 |
| Autism * Strengths Use | -0.02 | 0.02 | -0.06 | 0.14 |  | -0.01 | 0.12 | 0.00 | 0.20 |  | -0.05 | 0.10 | -0.05 | 0.24 |  | 0.11 | 0.12 | 0.08 | 0.18 |
| Autism * Age (years) | -0.04 | 0.02 | -0.08 | 0.63 |  | 0.10 | 0.11 | 0.05 | 0.21 |  | 0.06 | 0.09 | 0.03 | 0.26 |  | 0.05 | 0.10 | 0.03 | 0.23 |
| Autism * Sex | 0.87 | 0.41 | 0.09* | 1.24 |  | -3.74 | 2.05 | -0.09 | 0.68 |  | -1.55 | 1.79 | -0.05 | 0.27 |  | -0.89 | 2.04 | -0.02 | 0.21 |
| Autism * General Cognitive Ability | -0.06 | 0.06 | -0.04 | 0.20 |  | 0.23 | 0.29 | 0.04 | 0.22 |  | 0.06 | 0.26 | 0.01 | 0.24 |  | 0.09 | 0.29 | 0.02 | 0.22 |
| Model Statistics | *F*(11, 263) = 24.76  *p* <.001, *R^2^* = .51 | | | |  | *F*(11, 263) = 15.40  *p* <.001, *R^2^* = .39 | | | |  | *F*(11, 263) = 7.27  *p* <.001, *R^2^* = .23 | | | |  | *F*(11, 263) = 7.24  *p* <.001, *R^2^* = .23 | | | |

*Note.* Predictors are mean centered, with interaction terms calculated using the centered variables. Males = 1 and Females = 0 when coding participant sex.

* *p* < .05, ** *p* < .001

Table S5

*Regression Analysis Predicting Autism-Specific Quality of Life within the Autistic Group.*

| Predictors | *B* [95% CI] | *SE_B_* | *Β* | *sr^2^* | *p* | *BF_incl_* |
| --- | --- | --- | --- | --- | --- | --- |
| Strengths Knowledge | 0.02 [-0.03, 0.07] | 0.03 | 0.11 | .005 | .36 | 0.27 |
| Strengths Use | 0.05 [0.02, 0.07] | 0.01 | 0.39 | .057 | .002 | 140.40 |
| Age (years) | -0.03 [-0.05, 0.00] | 0.01 | -0.14 | .018 | .073 | 0.85 |
| Sex | 0.48 [-0.08, 1.03] | 0.28 | 0.13 | .016 | .094 | 0.59 |
| General Cognitive Ability | -0.01 [-0.09, 0.07] | 0.04 | -0.02 | .000 | .84 | 0.20 |
| Overall Model | *F*(5, 131) = 9.90, *p* <.001, *R^2^* = .27 | | | | |  |

*Note.* Males = 1 and Females = 0 when coding participant sex. Autism-Specific Quality of Life was a composite score calculated by summing standardised WHO-QOL-Disabilities module (Power & Green, 2010) and Autism-Specific QoL (ASQoL; McConachie et al., 2018) scores.

Table S6

*Regression Analysis Predicting Autism-Specific Quality of Life while Accounting for Autistic Traits*

| Predictors | *B* [95% CI] | *SE_B_* | *Β* | *sr^2^* | *p* | *BF_incl_* |
| --- | --- | --- | --- | --- | --- | --- |
| Strengths Knowledge | 0.04 [-0.01, 0.10] | 0.03 | 0.20 | .012 | .13 | 0.40 |
| Strengths Use | 0.03 [0.00, 0.06] | 0.02 | 0.29 | .027 | .027 | 40.47 |
| Autistic Traits | -0.05 [-0.09, -0.01] | 0.02 | -0.22 | .032 | .016 | 3.33 |
| Age (years) | -0.01 [-0.04, 0.02] | 0.02 | -0.06 | .003 | .44 | 0.43 |
| Sex | 0.40 [-0.17, 0.97] | 0.29 | 0.11 | .010 | .17 | 0.40 |
| General Cognitive Ability | 0.02 [-0.06, 0.10] | 0.04 | 0.04 | .002 | .60 | 0.24 |
| Autistic Traits * Strengths Knowledge | -0.00 [-0.01, 0.00] | 0.00 | -0.15 | .006 | .31 | 0.34 |
| Autistic Traits * Strengths Use | 0.00 [-0.00, 0.01] | 0.00 | 0.23 | .014 | .11 | 0.61 |
| Autistic Traits * Age (years) | 0.00 [-0,00, 0.00] | 0.00 | 0.02 | .000 | .84 | 0.27 |
| Autistic Traits * Sex | -0.03 [-0.11, 0.04] | 0.04 | -0.07 | .004 | .39 | 0.26 |
| Autistic Traits * General Cognitive Ability | -0.01 [-0.02, 0.00] | 0.01 | -0.10 | .007 | .24 | 0.45 |
| Overall Model | *F*(11, 125) = 5.40, *p* <.001, *R^2^* = .32 | | | | |  |

*Note.* Predictors are mean centered, with interaction terms calculated using the centered variables. Males = 1 and Females = 0 when coding participant sex. Autism-Specific Quality of Life was a composite score calculated by summing standardised WHO-QOL-Disabilities module (Power & Green, 2010) and Autism-Specific QoL (ASQoL; McConachie et al., 2018) scores.

**Supplementary References**

JASP Team (2021). *JASP (Version 0.16)* [Computer software].

Mathôt, S. (2017). Bayes like a Baws: Interpreting Bayesian Repeated Measures in JASP. *COGSCIdotNL.* <https://www.cogsci.nl/blog/interpreting-bayesian-repeated-measures-in-jasp>.

McConachie, H., Mason, D., Parr, J. R., Garland, D., Wilson, C., & Rodgers, J. (2018). Enhancing the validity of a quality of life measure for autistic people. *Journal of Autism and Developmental Disorders*, *48*(5), 1596–1611. <https://doi.org/10.1007/s10803-017-3402-z>

Power, M. J., & Green, A. M. (2010). Development of the WHOQOL disabilities module. *Quality of Life Research, 19*(4), 571–584.

Quintana, D. S., & Williams, D. R. (2018). Bayesian alternatives for common null-hypothesis significance tests in psychiatry: a non-technical guide using JASP. *BMC Psychiatry*, *18*(1), 178.

Rouder, J. N., Morey, R. D., Speckman, P. L., & Province, J. M. (2012). Default Bayes factors for ANOVA designs. *Journal of Mathematical Psychology*, *56*(5), 356-374.

Wagenmakers, E. J. (2007). A practical solution to the pervasive problems of *p* values. *Psychonomic Bulletin & Review*, *14*(5), 779-804.

Wagenmakers, E. J., Marsman, M., Jamil, T., Ly, A., Verhagen, J., Love, J., Selker, R., Gronau, Q. F., Šmíra, M., Epskamp, S., Matzke, D., Rouder, J. N., & Morey, R. D. (2018). Bayesian inference for psychology. Part I: Theoretical advantages and practical ramifications. *Psychonomic Bulletin & Review*, *25*(1), 35-57.

Wagenmakers, E. J., Wetzels, R., Borsboom, D., & Van Der Maas, H. L. (2011). Why psychologists must change the way they analyze their data: the case of psi: comment on Bem. *Journal of Personality and Social Psychology,* *100*(3), 426-432.
